# Aging-based molecular classification and score system in ccRCC uncovers distinct prognosis, tumor immunogenicity, and treatment sensitivity

**DOI:** 10.3389/fimmu.2022.877076

**Published:** 2022-08-11

**Authors:** Maoshu Zhu, Chaoqun Huang, Xinhong Wu, Ying Gu, Xiaoxu Hu, Dongna Ma, Weimin Zhong

**Affiliations:** ^1^ Department of Central Laboratory, the Fifth Hospital of Xiamen, Xiamen, China; ^2^ Department of Pharmacy, Xiang’an Hospital of Xiamen University, School of Medicine, Xiamen University, Xiamen, China; ^3^ Affiliated Primary School to Renmin University of China, Beijing, China; ^4^ Key Laboratory of the Ministry of Education for Coastal and Wetland Ecosystems, College of the Environment and Ecology, Xiamen University, Xiamen, China

**Keywords:** clear cell renal cell carcinoma, aging phenotypes, prognosis, tumor immunogenicity, treatment sensitivity

## Abstract

**Objective:**

Aging is a complex biological process and a major risk factor for cancer development. This study was conducted to develop a novel aging-based molecular classification and score system in clear cell renal cell carcinoma (ccRCC).

**Methods:**

Integrative analysis of aging-associated genes was performed among ccRCC patients in the TCGA and E-MTAB-1980 cohorts. In accordance with the transcriptional expression matrix of 173 prognostic aging-associated genes, aging phenotypes were clustered with the consensus clustering approach. The agingScore was generated to quantify aging phenotypes with principal component analysis. Tumor-infiltrating immune cells and the cancer immunity cycle were quantified with the ssGSEA approach. Immunotherapy response was estimated through the TIDE algorithm, and a series of tumor immunogenicity indicators were computed. Drug sensitivity analysis was separately conducted based on the GDSC, CTRP, and PRISM analyses.

**Results:**

Three aging phenotypes were established for ccRCC, with diverse prognosis, clinical features, immune cell infiltration, tumor immunogenicity, immunotherapeutic response, and sensitivity to targeted drugs. The agingScore was developed, which enabled to reliably and independently predict ccRCC prognosis. Low agingScore patients presented more undesirable survival outcomes. Several small molecular compounds and three therapeutic targets, namely, *CYP11A1*, *SAA1*, and *GRIK4*, were determined for the low agingScore patients. Additionally, the high agingScore patients were more likely to respond to immunotherapy.

**Conclusion:**

Overall, our findings introduced an aging-based molecular classification and agingScore system into the risk stratification and treatment decision-making in ccRCC.

## Introduction

Renal cell carcinoma (RCC) affects more than 400,000 people in the world annually (1). The age of diagnosis is around 60, and men are diagnosed twice as often as women (2). RCC has a few histological subtypes, with around 70% of individuals diagnosed with clear cell renal cell carcinoma (ccRCC) (1). Although ccRCC can be detected early and successfully treated with surgery or ablation regimens, over one-third of cases develop or progress to metastatic disease that is almost uniformly lethal (3). ccRCC is highly immune infiltrated, but there is extensive immune heterogeneity within and between patients (4). Immune checkpoint blockade (ICB) and combined strategies have favorably prolonged the survival of ccRCC patients (5–7). Tumor-infiltrating cells enable to influence the balance of antitumor immune response and immune escape in ccRCC, and T-cell exhaustion within the tumor microenvironment (TME) is responsible for the low response rate of ICB (4). Patients who respond to ICB present remarkable enrichment of tissue-resident T-cell populations, with enrichment of tumor-associated macrophages in resistant patients (4). Nevertheless, predicting which patients will respond to ICB remains a fundamental issue. Additionally, the drivers and resistors of ICB responses are still not fully elucidated.

Cancer is regarded as an aging-related degenerative malignancy, and aging is an independent risk factor of cancer (8). The mechanisms by which aging results in cancer progression remain being explored. Many aging-related cellular events (genomic instability, inflammatory response, immunity, etc.) are hallmarks of cancer (9). Despite the widespread study of the aging microenvironment in cancers, few studies focused on the overall characteristics of the transcriptional landscape of aging-associated genes in ccRCC. Previously, several aging-associated genes have been determined to be linked to unfavorable survival outcomes of RCC (9). Additionally, experimental evidence demonstrates that aging-associated genes participate in RCC progression. The molecular characteristics that reflect ccRCC ontogeny and development are being increasingly defined. Herein, on the basis of molecular and clinical information of ccRCC patients, we comprehensively evaluated aging phenotypes and their interactions with the tumor immune landscape. Three different aging phenotypes were characterized, which presented different prognosis and immunologic mechanisms, demonstrating the critical role of the aging process in remodeling tumor immune landscape in ccRCC individuals. Thereafter, aging phenotypes were individually quantified *via* generating the agingScore. Altogether, our findings might assist risk stratification and guide treatment decision-making for ccRCC.

## Materials and methods

### Publicly available datasets and processing

**Figure 1:**
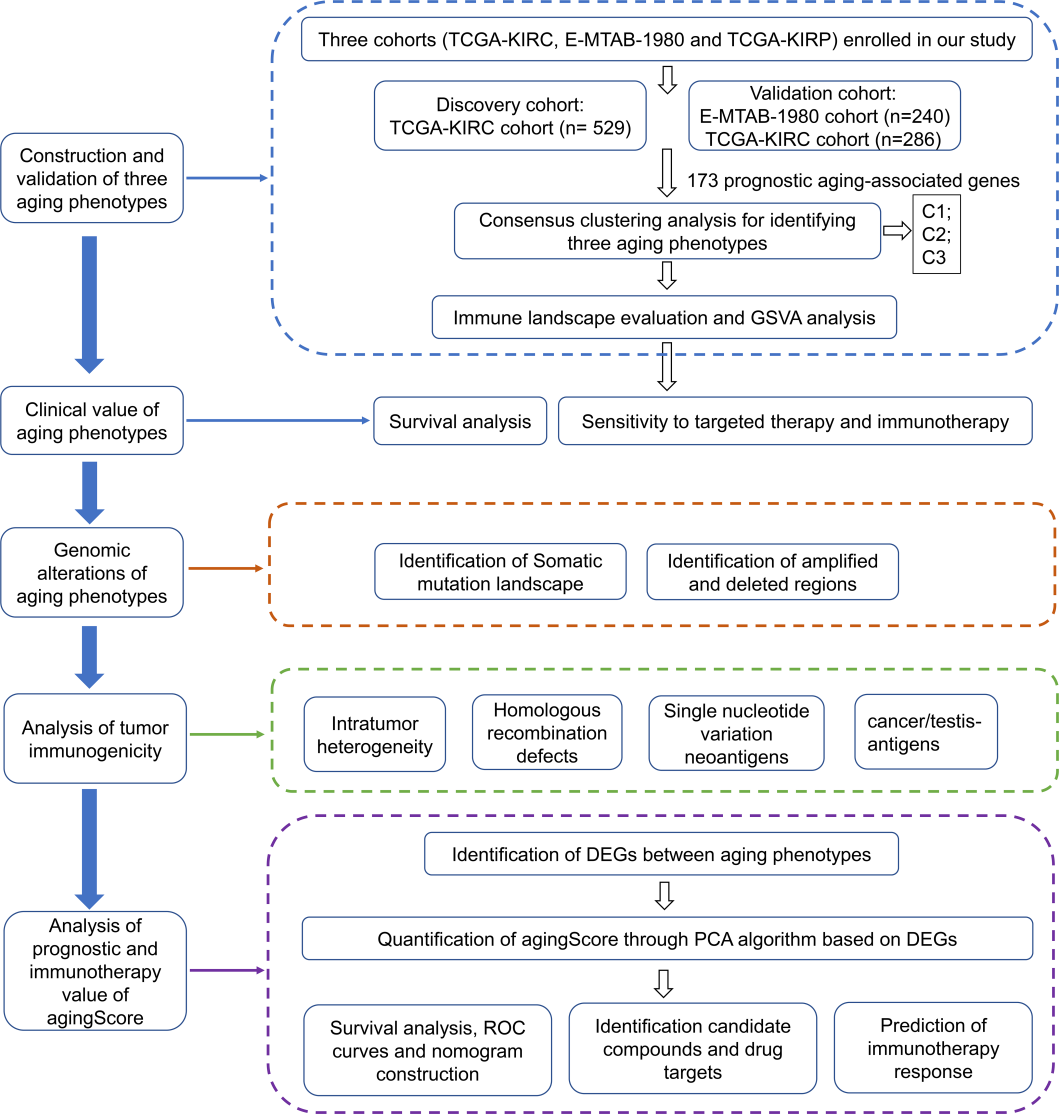
The workflow of our study.

For the discovery cohort, RNA sequencing (RNA-seq) data of The Cancer Genome Atlas-kidney renal clear cell carcinoma (TCGA-KIRC) cohort (*n* = 529) were obtained *via* the UCSC Xena project (https://xenabrowser.net/datapages/). For the verification cohorts, RNA-seq profiles of the TCGA-kidney renal papillary cell carcinoma (TCGA-KIRP) (*n* = 286) were also obtained from the UCSC Xena Portal, while the microarray data of the E-MTAB-1980 cohort (*n* = 240) were downloaded from the ArrayExpress database (https://www.ebi.ac.uk/arrayexpress/) (10). RNA-seq data were further transformed to log2 (TPM+1), which had higher similarity with microarray profiling and higher comparability between samples. The corresponding clinical information was collected from the UCSC and ArrayExpress databases (**Supplementary Table 1**). The microarray data were adjusted for background and normalized by quantile utilizing the robust multiarray average (RMA) approach from the affy package (11). For the TCGA-KIRC project, the somatic mutational data as well as the copy number alteration (CNA) data were acquired from the TCGA portal (https://portal.gdc.cancer.gov/). **Figure 1** depicts the work procedure of this study. We collected 307 aging-associated genes from the Human Ageing Genomic Resources (HAGR; https://genomics.senescence.info/), which is a collection of online resources for exploring the biology of human aging (12).

### Establishment of aging phenotypes

The optimum number of clusters was determined in the TCGA-KIRC cohort *via* the ConsensusClusterPlus package based on the expression profiling of prognostic aging-associated genes generated from the univariate Cox regression models (13). Eighty percent of the samples were subsampled in each iteration, and each subsample was divided into at most *k* (maximum *k* value = 9) groups *via* the *k*-means algorithm through the Euclidean distance. This analysis was repeated 1,000 times. Thereafter, the perfect clustering result was determined by considering consistent cumulative distribution function (CDF) graphs. Afterward, the results were illustrated as consensus matrix heatmaps generated by the heatmap package. The reproducibility of the clusters was evaluated in the E-MTAB-1980 cohort.

### Functional and pathway enrichment analysis

The single-sample gene set enrichment analysis (ssGSEA) algorithm from the gene set variation analysis (GSVA) software (14) was implemented to ascertain the hallmark pathways and evaluate the differences in biological significance among aging phenotypes. The hallmark gene set was collected from the Molecular Signatures Database (MSigDB) to run the GSVA. Through the clusterProfiler package (15), functional annotation of aging phenotype-associated genes was carried out. *P <*0.05 was regarded as significant enrichment in Gene Ontology and Kyoto Encyclopedia of Genes and Genomes (KEGG). The known pathways were acquired, consisting of epithelial–mesenchymal transition (EMT1, 2, 3), immune checkpoint, antigen processing machinery, CD8^+^ T effector signature, angiogenesis signature, and pan-fibroblast TGFβ response signature (pan-FTBRS) (16–18). The activities of the above biological processes were computed *via* the ssGSEA approach.

### Generation of tumor-infiltrating immune cells

Through the ssGSEA, the infiltrations of 28 immune cell types were quantified in accordance with the 782 metagenes utilizing the GSVA package (14). Tumor purity as well as stromal and immune scores was computed *via* the ESTIMATE package with default parameters (19).

### Cancer immunity cycle

The cancer immunity cycle can reflect the anti-tumor immune response and comprises seven steps, as previously described (20). The activities of the above steps were quantified *via* the ssGSEA approach.

### Immunotherapy response

Through the tumor immune dysfunction and exclusion (TIDE) algorithm, the response to immunotherapy was predicted in accordance with the tumor immune escape mechanisms: inducing T-cell dysfunction within tumors with enhanced infiltrations of cytotoxic T lymphocytes (CTLs) as well as preventing the infiltrations of T cells within tumors with reduced CTLs [21]. The expression similarity between phenotypes and the patients who differently responded to immunotherapy was evaluated with the Subclass Mapping (SubMap) approach that employed the GSEA algorithm to infer the commonality between groups (21). *P <*0.05 suggested a significant similarity between groups.

### Drug sensitivity analysis

Through the pRRophetic package (22), a ridge regression analysis was conducted in accordance with the Genomics of Drug Sensitivity in Cancer (GDSC) cell line expression data (23). The half maximal inhibitory concentration (IC_50_) value was estimated to reflect the sensitivity to agents. The expression profiling and somatic mutational data of human cancer cell lines (CCLs) were retrieved from the Cancer Cell Line Encyclopedia (CCLE) database (https://portals.broadinstitute.org/ccle/) (24). CERES scores can be utilized for measuring the dependency of target genes in certain CCLs. A negative score represents that the cell line grows slower when the specific gene is knocked out, and a positive score represents that the cell line grows faster when the specific gene is knocked out. The CERES scores of CRISPR-knockdown screening of over 18,000 genes across over 700 cell lines were acquired from the dependency map (DepMap) database (https://depmap.org/portal/). Drug sensitivity data of CCLs were required from the Cancer Therapeutics Response Portal (CTRP; https://portals.broadinstitute.org/ctrp) containing 481 agents over 835 CCLs as well as from the PRISM project (https://depmap.org/portal/prism/) containing 1,448 agents over 482 CCLs. The two datasets offer the area under the dose–response curve (AUC) measures of drug sensitivity. A lower AUC value indicates a higher sensitivity to an agent. Since the CCLs in the two projects were required from the CCLE project, expression profiling in CCLE was utilized for further CTRP and PRISM analysis.

### Analysis of single-nucleotide polymorphisms and CNAs

The maftools package was applied for analyzing somatic variants, and overall mutation status was illustrated across three phenotypes (25). Through the GISTIC (version 2.0) software, the amplified and deleted CNAs in the tumor were quantified with the input of ‘‘SNP6” files (26).

### Evaluation of tumor immunogenicity indicators

Immune checkpoints with therapeutic potential were collected from the study of Auslander et al. (27). Single nucleotide variation (SNV) neoantigens were calculated *via* NetMHCpan (version 3.0) (28) in accordance with human leukocyte antigen (HLA) types required from RNA-seq utilizing OptiType (version 1.2) (29). Tumor mutation burden (TMB) was quantified following the total count of non-synonymous mutations (30). Homologous recombination defects score was computed through three DNA-based genomic instability: large (>15 Mb) non-arm-level regions with loss of heterozygosity, telomeric allelic instability, and large-scale state conversion with breaks between adjacent segments >10 Mb (31). Intratumor heterogeneity and cancer/testis antigens (CTAs) were also involved.

### Analysis of aging phenotype-associated genes

The limma package was applied for determining the differentially expressed genes (DEGs) between phenotypes (32). The threshold was set as adjusted *P <*0.05 as well as at least 1.5-fold changes in expression. The Venn plot directly depicted the number of DEGs among phenotypes.

### Generation of the aging gene signature

For quantifying the aging phenotypes of individual patients, a scoring system was generated for assessing all individuals, named as agingScore. Univariate Cox regression analysis was conducted to determine the prognostic implication of DEGs. The DEGs with *P <*0.05 were utilized to compute the agingScore *via* the PCA algorithm. The principal components (PCs) PC1 and PC2 acted as the scoring system (33, 34). The formula of the agingScore was as follows: $\text{aging Score}=\sum_{i}^{j}PCi+PCj$ where *i* and *j* indicate the ranking and the total number of the prognostic DEGs, respectively. The advantage of this approach is to focus the score on the set with the largest well-related (or anti-correlated) genes in the set while reducing the contribution of genes that are not tracked with other set members.

### Analysis of post-transcriptional features correlated to agingScore

In the TCGA-KIRC cohort, differentially expressed miRNAs or mRNAs were screened between high and low agingScore groups in accordance with FDR <0.05 and at least 1.5-fold changes. The targeted mRNAs were then predicted through the miRbase database (http://www.mirbase.org/) (35). Analysis of KEGG pathways enriched by the miRNA-targeted mRNAs was carried out.

### Statistical analysis

The dissimilarity of the clusters was verified through principal component analysis (PCA). Student’s *t*-test or the Wilcoxon rank sum test was applied to evaluate the difference between two groups, while one-way analysis of variance or the Kruskal–Wallis test was implemented to estimate the difference between three groups. The Benjamini–Hochberg approach was implemented to correct multiple tests. Kaplan–Meier analysis was implemented to compare the overall survival (OS), disease-free survival (DFS), disease-specific survival (DSS), and progression-free survival (PFS) between groups utilizing survival and survminer packages. The difference in OS was computed with the log-rank test. The receiver operating characteristic (ROC) curves of OS, DFS, DSS, and PFS were conducted and the AUC values were calculated with the survivalROC package. Univariate Cox regression analysis was implemented to identify the significant associations of agingScore and clinical features with OS, DFS, DSS, and PFS. Hazard ratio (HR), 95% confidence interval (CI), and the *P*-value of each variable were separately computed. Multivariable Cox regression analysis was utilized to assess whether agingScore was independent of other clinical features. In accordance with the multivariate Cox regression results, a nomogram was generated to provide a visualized risk prediction after each factor was assigned a score *via* the rms package. The time-dependent concordance index (C-index) was computed with the pec package. A calibration diagram was drawn to estimate the calibration capacity of the nomogram. Correlation analysis was implemented with Pearson’s or Spearman’s correlation test. All statistical *P*-values were two-sided, with *P <*0.05 as statistically significant. All analyses were conducted with the R software (version 4.0.2).

## Results

### The landscape of prognosis and clinical features of the three aging phenotypes in ccRCC

**Figure 2:**
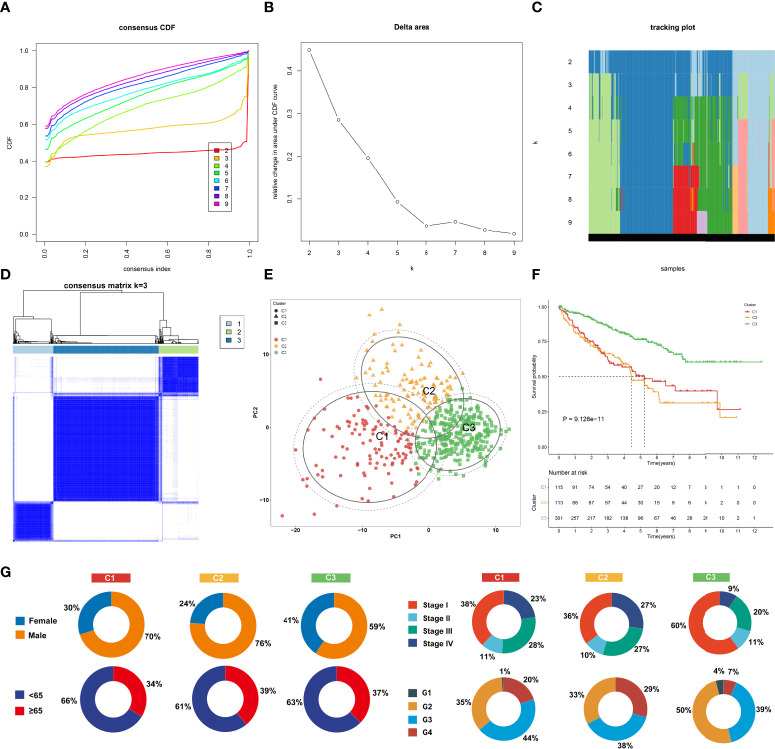
The landscape of prognosis and clinical features of the three aging phenotypes in The Cancer Genome Atlas-kidney renal clear cell carcinoma (TCGA-KIRC) cohort. **(A)** The cumulative distribution functions (CDFs) of the consensus matrix for *k* = 2~9 marked by colors. **(B)** Relative alterations in area under the CDFs for *k* = 2~9. **(C)** Tracking plot for the classification of the TCGA-KIRC dataset into diverse subtypes for *k* = 2~9. **(D)** Classification of the TCGA-KIRC cohort into three clusters when *k* = 3. **(E)** PCA of the RNA expression profiling of prognostic aging-associated genes. **(F)** Kaplan–Meier analysis for overall analysis (OS) among the three phenotypes. **(G)** Pie plots of the distribution of clinical features across the three phenotypes.

Among 307 aging-associated genes, univariate Cox regression determined 173 genes significantly linked to clinical outcomes among 529 ccRCC patients in the TCGA-KIRC cohort (**Supplementary Table 2**), which were included for consensus clustering analysis. Through applying the ConsensusClusterPlus approach, three aging phenotypes were achieved, termed as C1, C2, and C3 in accordance with the selection of *k* = 3 as the optimal *k* value (**Figures 2A–D**). Thereafter, PCA affirmed the remarkable difference among the three phenotypes (**Figure 2E**). Patients with the C3 phenotype presented the best OS outcomes, followed by C1 and C2 (**Figure 2F**). **Figure 2G** illustrates the distribution of clinical features among the three phenotypes. Because the E-MTAB-1980 cohort possessed relatively complete prognostic information as well as a large sample size, it was employed for validating the repeatability of this classification. Similarly, consensus clustering analysis was implemented on the cohort, and the three different aging phenotypes were clearly clustered (**Supplementary Figures 1A–E**). The remarkable difference in OS outcomes was noted among the phenotypes (**Supplementary Figure 1F**), affirming the reliability of this classification for ccRCC.

### The immune landscape of the three aging phenotypes

**Figure 3:**
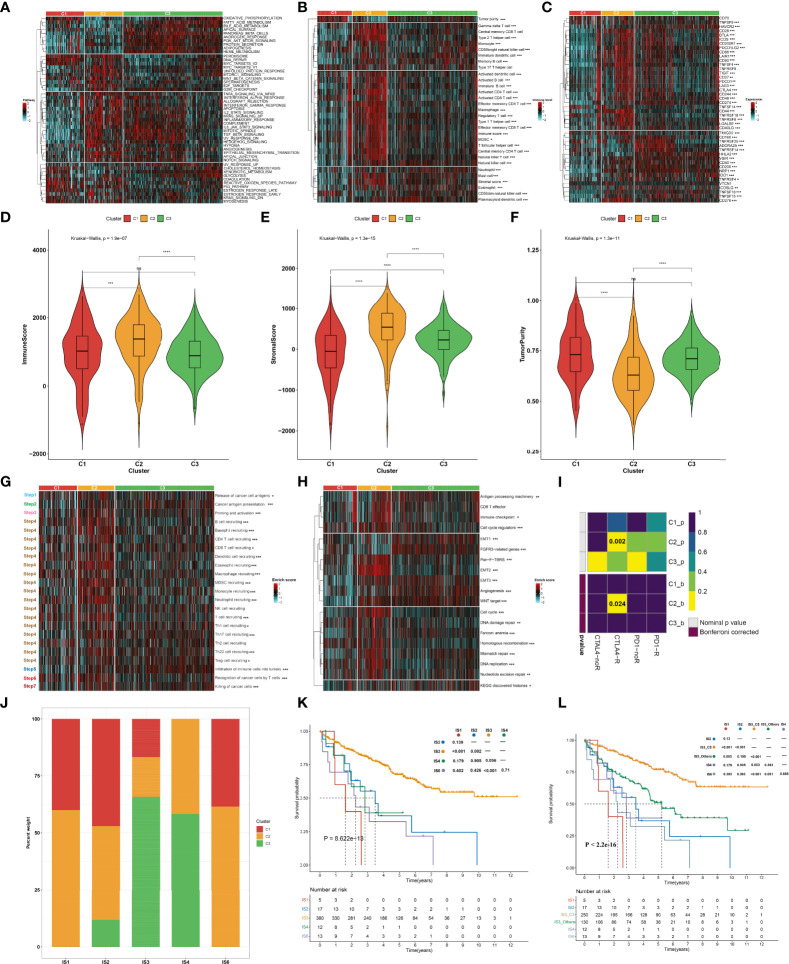
Immune landscape and antitumor immune responses of the three aging phenotypes in the TCGA-KIRC cohort. **(A)** Heatmap depicting the activities of hallmark pathways across the three aging phenotypes. Activated and inhibited hallmark pathways are colored by red and cyan, respectively. **(B)** Heatmap of the relative abundance of tumor-infiltrating immune cell populations across the three aging phenotypes. The high and low enrichment levels are colored red and cyan, respectively. **(C)** Heatmap of the RNA expression of immune checkpoint molecules across the three aging phenotypes. The high and low expression levels of immune checkpoints are marked by red and cyan, respectively. **(D–F)** Box plots of the differences in **(D)** immune and **(E)** stromal scores as well as **(F)** tumor purity across the three aging phenotypes. **(G)** Heatmap of the activation states of the seven steps within the cancer immunity cycle across the three aging phenotypes. Activated and inhibited steps are colored red and cyan, respectively. **(H)** Heatmap of the activities of known biological processes across the three aging phenotypes. Activated or inactivated processes are colored red or cyan. **(I)** Prediction of the response to anti-PD-1 and anti-CTLAL4 among the three aging phenotypes. R, response; noR, non-response. **(J)** Distribution of the three aging phenotypes across known immune subtypes. **(K)** Kaplan–Meier analysis for OS of the TCGA-KIRC cohort stratified by immune subtypes. **(L)** Kaplan–Meier analysis for OS of the TCGA-KIRC cohort stratified by immune subtypes and aging phenotypes. Ns, no significance; *P < 0.05; **P < 0.01; ***P < 0.001; ****P < 0.0001.

To uncover the molecular mechanisms underlying the aging phenotypes, we focused on the TCGA-KIRC dataset that had relatively complete omics data and clinical characteristics. The activity status of the hallmark pathways was firstly computed across the three aging phenotypes. In **Figure 3A**, oxidative phosphorylation, peroxisome, reactive oxygen species pathway, and DNA repair presented relatively high activities in the C1 phenotype; the C2 phenotype had relatively high activities of immune pathways (IL2–STAT5 signaling, inflammatory response, complement, etc.) as well as stromal pathways (WNT beta-catenin signaling, angiogenesis, etc.); and the C3 phenotype presented features of activation of metabolism pathways (fatty acid metabolism, bile acid metabolism, etc.). Extrinsic immune evasion mechanisms were evaluated in accordance with the absence of leukocytes as well as the presence of immunosuppressive cell populations. As illustrated in **Figure 3B**, the C1 phenotype presented deficient immune cell infiltrations and immune-mediated elimination, which can be characterized as the immune-deserted type; the C2 phenotype was recognized as the immune-excluded type which had relatively high stromal score and infiltrations of immunosuppressive cells [type 1 helper cells, type 2 helper cells, regulatory T cells, myeloid-derived suppressor cells (MDSCs), T follicular helper cells, etc.]; and the C3 phenotype was recognized as immune-inflamed characterized by the immune-active cells (CD4^+^ T cells, CD8^+^ T cells, etc.). Therefore, we speculated that the C1 and C3 phenotypes probably reflected the deficiency of recruitment or activation of innate immune cell populations, thereby triggering the failure of adaptive antitumor immune responses. Moreover, the C2 phenotype had a higher expression of co-stimulatory and co-inhibitory immune checkpoint molecules in comparison to the other phenotypes (**Figure 3C**), indicating that the C2 phenotype might upregulate the immune checkpoint molecules to escape the immune elimination following immune activation. Through the ESTIMATE approach, we estimated the overall levels of immune and stromal cells as well as tumor purity. Not surprisingly, the C2 phenotype presented higher immune and stromal scores as well as lower tumor purity compared with the other phenotypes (**Figures 3D–F**).

### Antitumor immune responses of the three aging phenotypes

The activities of the cancer immunity cycle were computed to reflect antitumor immune responses. In **Figure 3G**, most steps within the cancer immunity cycle showed higher activity status in the C2 phenotype in comparison to the other phenotypes. It was also noted that the C2 phenotype was characterized by stromal activation (EMT, pan-F-TBRS, etc.) as well as cell cycle progression (cell cycle, DNA replication, etc.), as illustrated in **Figure 3H**. It was predicted that the C2 phenotype responded to anti-CTAL4 therapy (**Figure 3I**). A previous study has defined six major immune subtypes (ISs) across pan-cancer in TCGA: IS1 (wound healing), IS2 (IFN-γ dominant), IS3 (inflammatory), IS4 (lymphocyte depleted), IS5 (immunologically quiet), and IS6 (TGF-β dominant) (36). We sought to comprehend the interaction of aging phenotypes with immune subtypes. Overall, the IS1, IS2, and IS6 subtypes tended to have more C1 and C2 aging phenotypes, with more C3 aging phenotype in the IS3 and IS4 subtypes (**Figure 3J**). Among five immune subtypes, ccRCC patients in the IS3 subtype presented the best OS outcomes, with the worst outcomes for those in the IS1 subtype (**Figure 3K**). Interestingly, the C3 patients were significantly enriched in the IS3 subtype (16.84% in C1, 17.37% in C2, 65.79% in C3). Although both C3 and IS3 showed a favorable prognosis, nearly 34% of IS3 patients had C1 and C2 phenotypes, which resulted in a poor prognosis. Thus, we explored whether aging-related clusters could further categorize patients into distinct survival groups. By performing a log-rank test, IS3 belonging to the C3 phenotype showed a more favorable prognosis compared to the other phenotypes (**Figure 3L**). These results indicated that aging-related genes could provide an additional characterization from a preexisting molecular classification of ccRCC.

### Sensitivity to known targeted drugs of the three aging phenotypes

**Figure 4:**
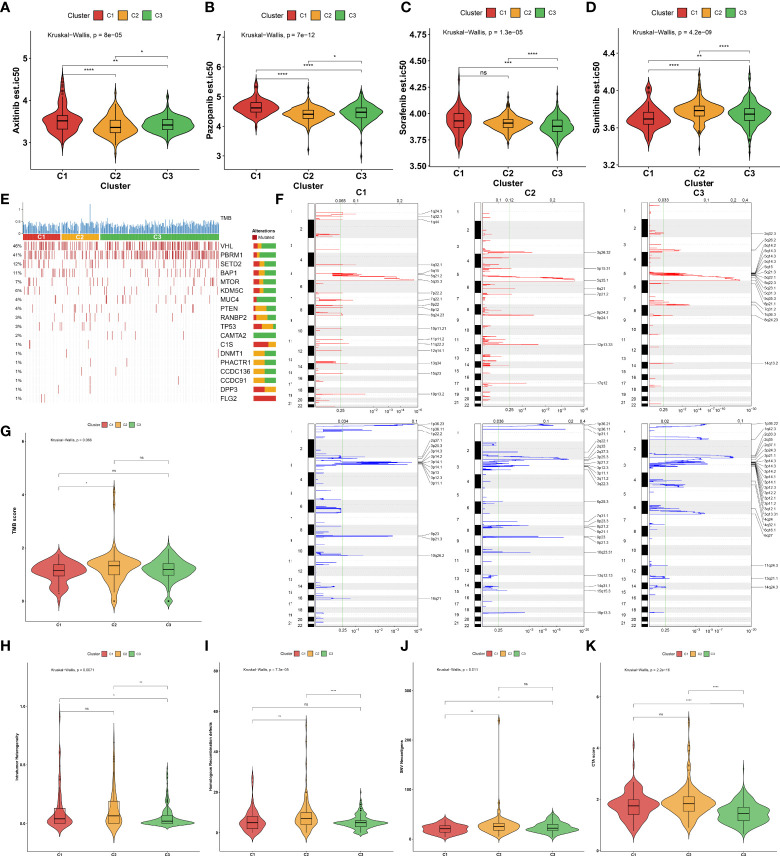
Sensitivity to known targeted drugs, genomic alterations, and intrinsic immune evasion mechanisms of the three aging phenotypes in the TCGA-KIRC cohort. **(A–D)** Box plots of the differences in sensitivity to **(A)** axitinib, **(B)** pazopanib, **(C)** sorafenib, and **(D)** sunitinib across the three aging phenotypes. **(E)** Waterfall plot of tumor somatic mutation across the three aging phenotypes. The top bar plot shows TMB. Each column represents an individual patient. Numbers and bar graphs depict the mutational frequencies as well as the proportions of mutation types. **(F)** Somatic copy number alterations across the three aging phenotypes. The *y*-axis indicates the chromosome positions, while the *x*-axis depicts the focal deletion or amplification identified by a horizontal blue or red bar. The green line indicates the significance threshold of FDR <0.25. (**G–K**) Box plots of the differences in **(G)** TMB score, **(H)** intratumor heterogeneity, **(I)** homologous recombination defects, **(J)** SNV neoantigens, and **(K)** CTA score across the three aging phenotypes. Ns, no significance; *P < 0.05; **P < 0.01; ***P < 0.001; ****P < 0.0001.

Considering that targeted treatment remains the preferred therapeutic regimen against advanced RCC, the sensitivity to axitinib, pazopanib, sorafenib, and sunitinib across the three aging phenotypes was assessed on the basis of the GDSC cell line expression data. The C2 phenotype presented higher sensitivity to axitinib (**Figure 4A**) and pazopanib (**Figure 4B**), the C3 phenotype was more sensitive to sorafenib (**Figure 4C**), and the C1 phenotype had higher sensitivity to sunitinib (**Figure 4D**).

### Genomic alterations of the three aging phenotypes

The somatic mutation landscape was determined in the TCGA-KIRC cohort. Among the three aging phenotypes, *VHL* (48%) and *PBRM1* (41%) were the most frequent mutated genes (**Figure 4E**). Additionally, the mutated genes were evenly distributed across the three aging phenotypes. Further analysis was conducted to delineate the significant focal copy number alterations *via* GISTIC 2.0. **Figure 4F** illustrates the distinct focal amplifications and deletions of CNVs in each phenotype. In comparison to the C1 phenotype, a higher TMB score was investigated in the C2 phenotype (**Figure 4G**). The diverse genomic alteration preferences across the three phenotypes might result in distinct ccRCC progression.

### Intrinsic immune evasion mechanisms of the three aging phenotypes

The intrinsic immune evasion mechanisms underlying the three aging phenotypes were evaluated in accordance with a series of indicators associated with tumor immunogenicity. The C3 phenotype had a higher intratumor heterogeneity compared with the C1 and C2 phenotypes (**Figure 4H**). Genomic instability was evaluated according to homologous recombination defects. The C2 phenotype presented higher homologous recombination defects in comparison to the other phenotypes (**Figure 4I**). In contrast to the C1 phenotype, increased SNV neoantigens were investigated in the C2 and C3 phenotypes (**Figure 4J**). Moreover, the C3 phenotype had a lower overall expression of CTAs in comparison to the C1 and C2 phenotypes (**Figure 4K**). Overall, the above elements associated with tumor immunogenicity had remarkable differences in the three aging phenotypes.

### Identification of aging phenotype-associated genes and generation of an agingScore system for ccRCC

**Figure 5:**
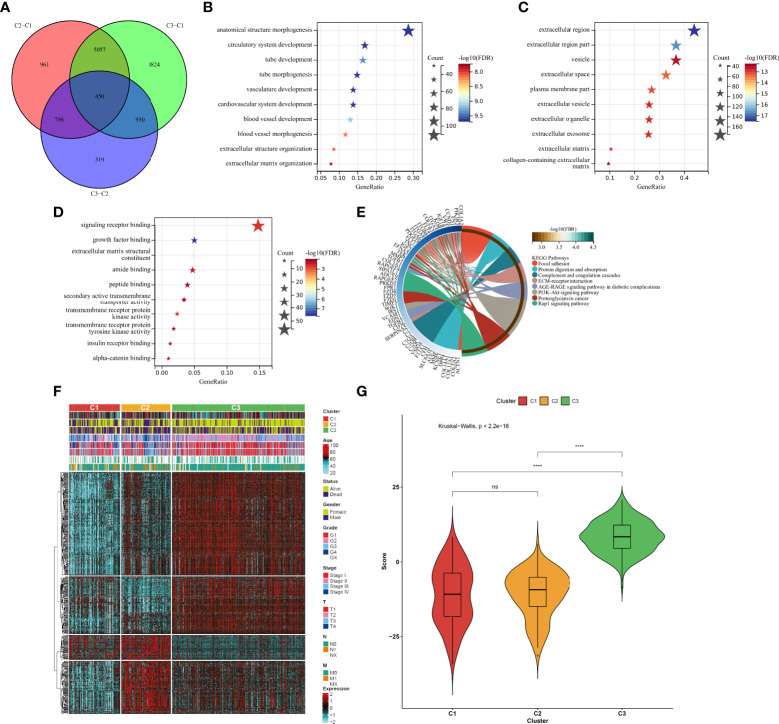
Identification of the aging phenotype-associated genes and generation of an agingScore system for ccRCC. **(A)** Venn diagram for the aging phenotype-associated genes *via* comparing the aging phenotypes. **(B–D)** The major **(B)** biological processes, **(C)** cellular components, or **(D)** molecular components enriched by aging phenotype-associated genes. **(E)** The major KEGG pathways enriched by aging phenotype-associated genes. **(F)** Distribution of the expression patterns of the aging phenotype-associated genes and clinical features across the three aging phenotypes. **(G)** Box plots of the distribution of agingScore among the three aging phenotypes. Ns, no significance; ****P < 0.0001.

**Figure 6:**
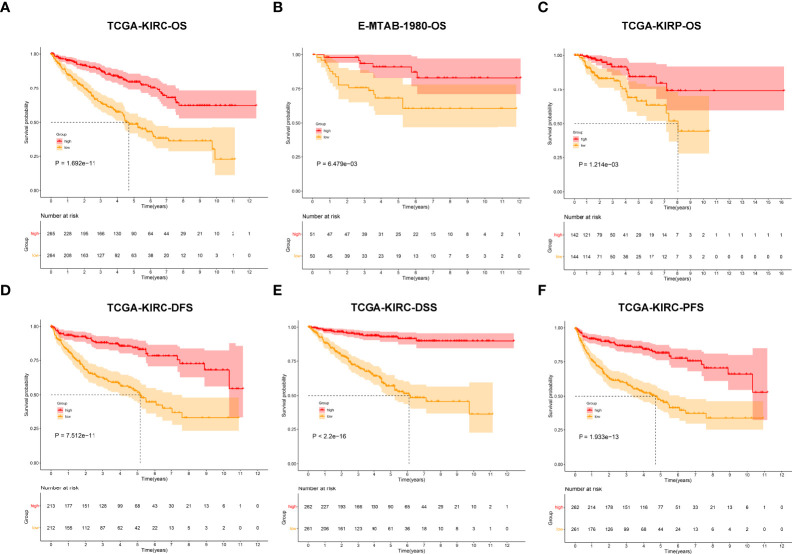
Analysis of the prognostic implication of the agingScore. **(A–C)** Kaplan–Meier analysis for OS between the high and low agingScore groups in the **(A)** TCGA-KIRC cohort, **(B)** E-MTAB-1980 cohort, and **(C)** TCGA-KIRP cohort. (**D–F**) Kaplan–Meier analysis for **(D)** DFS, **(E)** DSS, and **(F)** PFS between the high and low agingScore groups in the TCGA-KIRC cohort.

In total, 450 aging phenotype-associated genes were identified by comparing the differential expression between aging phenotypes (**Figure 5A**; **Supplementary Table 3**). Further analysis demonstrated their roles in vessel morphogenesis and development, extracellular components, and signaling receptor binding (**Figures 5B–D**). Additionally, these aging phenotype-associated genes were involved in mediating tumorigenic pathways (ECM–receptor interaction, PI3K–Akt signaling pathway, proteoglycans in cancer, Rap1 signaling pathway, etc.; **Figure 5E**). As illustrated in **Figure 5F**, there was widespread heterogeneity in the aging phenotype-associated genes among the three aging phenotypes. Univariate Cox regression determined 261 prognostic aging phenotype-associated genes in the TCGA-KIRC cohort (**Supplementary Table 4**). With the PCA approach, we computed an agingScore system for ccRCC. The C3 phenotype presented a higher agingScore in comparison to the C1 and C2 phenotypes (**Figure 5G**). In accordance with the median value of agingScore, we stratified the TCGA-KIRC, E-MTAB-1980, or TCGA-KIRP cohort into high and low agingScore populations. A high agingScore presented more favorable OS outcomes in comparison to a low agingScore in the TCGA-KIRC cohort (**Figure 6A**), E-MTAB-1980 cohort (**Figure 6B**), and TCGA-KIRP cohort (**Figure 6C**). Additionally, in the TCGA-KIRC cohort, a high agingScore was linked to better DFS (**Figure 6D**), DSS (**Figure 6E**), and PFS (**Figure 6F**), affirming the prognostic implication of the agingScore in ccRCC.

### Validation of the predictive performance of the agingScore in ccRCC prognosis

**Figure 7:**
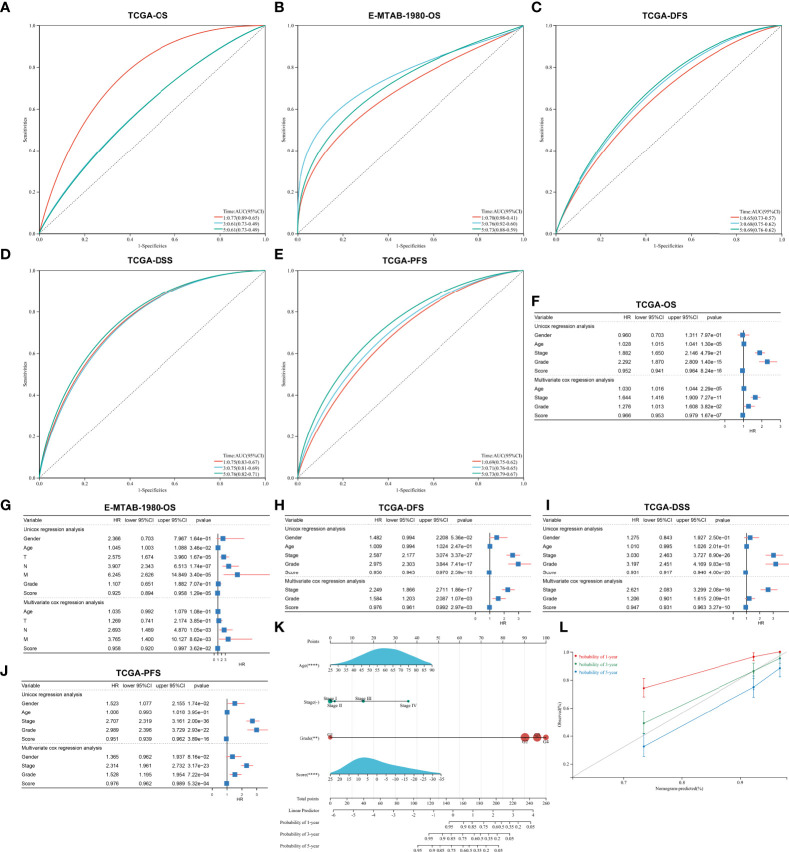
Validation of the predictive performance of the agingScore and generation of an agingScore-based nomogram for ccRCC prognosis. **(A, B)** ROC diagram of the 1-, 3-, and 6-year OS in the TCGA-KIRC cohort and the E-MTAB-1980 cohort. **(C–E)** ROC curves of the 1-, 3-, and 5-year DFS, DSS, and PFS in the TCGA-KIRC cohort. **(F, G)** Univariate and multivariate Cox regression models of agingScore as well as clinical features with OS in the TCGA-KIRC and E-MTAB-1980 cohorts. **(H–J)** Univariate and multivariate Cox regression analyses of agingScore and clinical features with DFS, DSS, and PFS in the TCGA-KIRC cohort. **(K)** Generation of an agingScore-based nomogram comprised of independent predictive indicators in the TCGA-KIRC cohort. (**L**) Calibration diagram for the nomogram-estimated and actual 1-, 3-, and 5-year OS in the TCGA-KIRC cohort.

The ROC diagram was drawn to verify the efficacy of this agingScore in predicting ccRCC prognosis. The AUC values of the 1-, 3-, and 5-year OS were 0.77, 0.61, and 0.61 in the TCGA-KIRC cohort and 0.70, 0.76, and 0.73 in the E-MTAB-1980 cohort (**Figures 7A, B**). Moreover, in the TCGA-KIRC cohort, the AUC values of the 1-, 3-, and 5-year DFS were 0.65, 0.68, and 0.69, respectively (**Figure 7C**); the AUC values of the 1-, 3-, and 5-year DSS were 0.75, 0.75, and 0.76, respectively (**Figure 7D**); and the AUC values of the 1-, 3-, and 5-year PFS were 0.69, 0.71, and 0.73, respectively (**Figure 7E**). Overall, the agingScore was a reliable prognostic indicator of ccRCC. In both the TCGA-KIRC and E-MTAB-1980 cohorts, the agingScore was an independent protective factor of ccRCC patients’ OS (**Figures 7F, G**). Additionally, the agingScore was independently predictive of ccRCC patients’ DFS, DSS, and PFS (**Figures 7H–J**).

### Construction of an agingScore-based nomogram for ccRCC prognosis

To facilitate the clinical application of the agingScore, we conducted a personalized nomogram model. This nomogram for OS prediction was conducted by incorporating the following independent prognostic factors: age, stage, grade, and agingScore. In **Figure 7K**, the 1-, 3-, and 5-year OS for ccRCC individuals from the TCGA-KIRC cohort can be evaluated by this personalized nomogram model (C-index = 0.782). As illustrated in the calibration diagram, a favorable overlap was found between the nomogram-predicted and the observed 1-, 3-, and 5-year OS of ccRCC patients (**Figure 7L**). Overall, the nomogram had a good prediction performance in ccRCC prognosis.

### Identification of agingScore-related candidate compounds and drug targets

**Figure 8:**
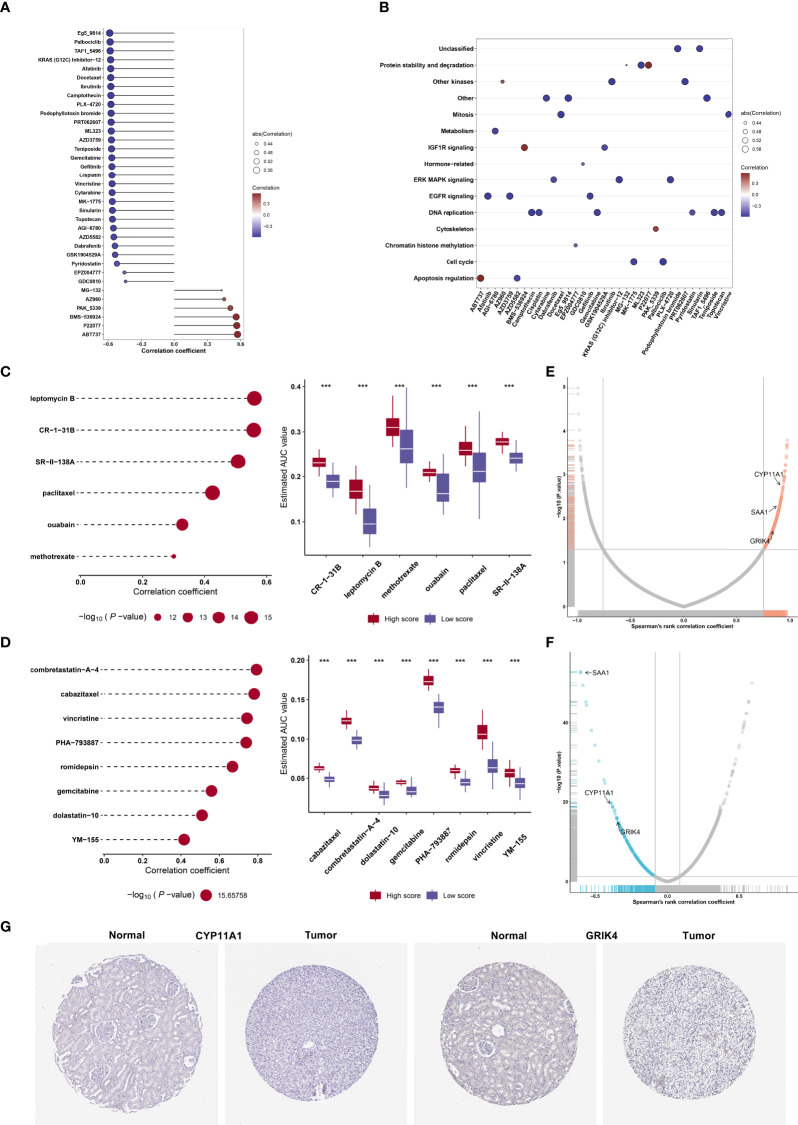
Identification of agingScore-related candidate compounds and drug targets. **(A)** Spearman’s correlation analysis of GDSC-derived compounds and agingScore. **(B)** Mechanisms involving GDSC-derived compounds. **(C)** Spearman’s correlation analysis of six CTRP-derived compounds and agingScore as well as differential analysis of drug responses between the high and low agingScore groups. **(D)** Spearman’s correlation analysis of eight PRISM-derived compounds and agingScore as well as differential analysis of drug responses between groups. A lower AUC value implies higher drug sensitivity. ***P < 0.001. **(E)** Volcano plot of Spearman’s correlation between agingScore and CERES score of drug targets. The red dot represents the significantly positive correlation (*P* < 0.05, and Spearman’s correlation coefficient>0.82). **(F)** Volcano plot of Spearman’s correlation between agingScore and protein expression of drug targets. The blue dot indicates a significant negative correlation (*P* < 0.05, and Spearman’s correlation coefficient ≤ 0.1). **(G)** Immunohistochemistry of the expression of *CYP11A1* and *GRIK4* in normal and kidney cancer tissues. Bar, 200 μm.

On the basis of the GDSC cell line data, we computed the associations of the AUCs of the GDSC-derived compounds with agingScore. **Figure 8A** illustrates the agingScore-related GDSC-derived compounds. The above compounds were involved in the tumorigenic pathways (apoptosis regulation, DNA replication, cell cycle, EGFR signaling, etc.; **Figure 8B**). The AUCs of six CTRP-derived compounds (leptomycin B, CR-1-31B, SR-II-138A, paclitaxel, ouabain, and methotrexate) were positively correlated to agingScore (**Figure 8C**). The low agingScore group presented lower AUCs of the above compounds in comparison to the high agingScore group, demonstrating that patients with low agingScore were more likely to be sensitive to the above compounds. Additionally, we determined the positive associations of the AUCs of eight PRISM-derived compounds (combretastatin-A-4, cabazitaxel, vincristine, PHA-793887, romidepsin, gemcitabine, dolastatin-10, and YM-155) with agingScore (**Figure 8D**). Lower AUCs were found in the low agingScore group, indicating higher sensitivity to the above compounds. Through Spearman’s correlation analysis between the CERES score of drug targets and agingScore, 261 targets were screened (*P* < 0.05, and correlation coefficient > 0.82; **Figure 8E**). Additionally, Spearman’s correlation analysis of druggable protein expression with agingScore was carried out. As a result, 242 protein targets were determined based on *P <*0.05 and correlation coefficient ≤0.1 (**Figure 8F**). Three genes, namely, *CYP11A1*, *SAA1*, and *GRIK4*, were determined as potential therapeutic targets through the above analysis, indicating that suppressing the functions of the above genes in low agingScore individuals could present desirable therapeutic effects. Moreover, *CYP11A1* and *GRIK4* expression was validated in normal and kidney tissues by immunohistochemistry from the Human Protein Atlas (**Figure 8G**).

### Association between agingScore and antitumor immune responses

**Figure 9:**
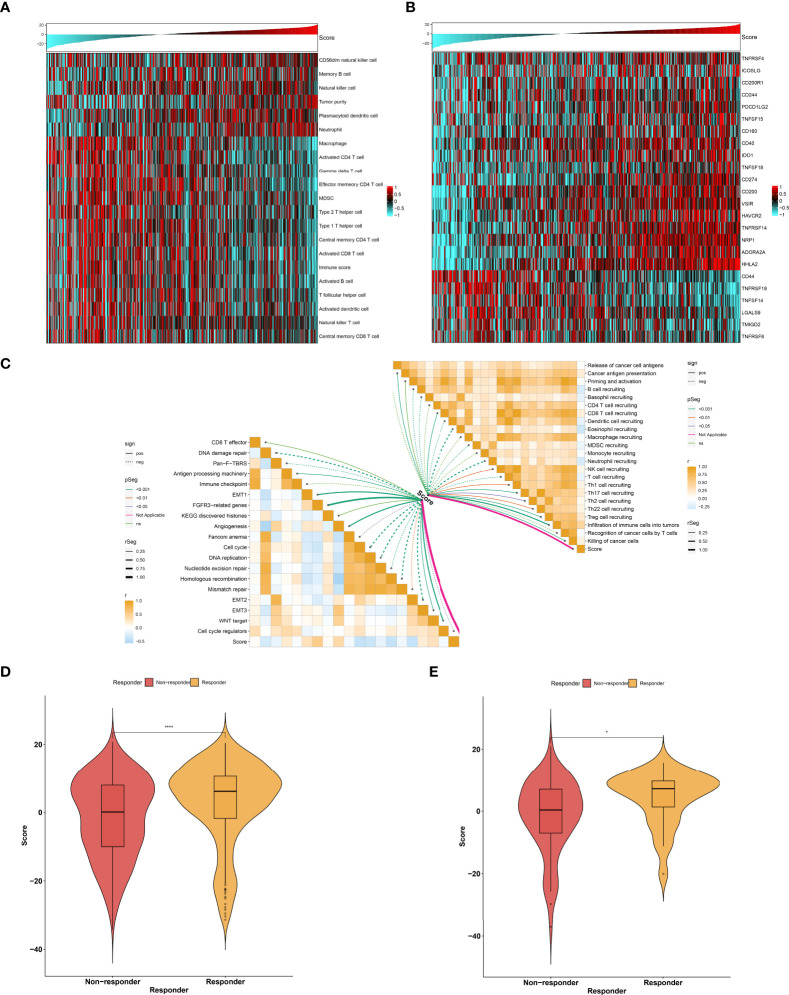
Association between agingScore and antitumor immune responses. **(A)** Landscape of immune cell infiltrations in the high as well as low agingScore groups in the TCGA-KIRC dataset. **(B)** Distribution of the expression of immune checkpoint molecules in the high and low agingScore groups in the TCGA-KIRC cohort. **(C)** Association of agingScore with activities of cancer immunity cycle and known biological processes in the TCGA-KIRC dataset. Solid lines represent positive correlations, while dashed lines represent negative correlations. **(D)** Prediction of response to immunotherapy in the TCGA-KIRC dataset. **(E)** Validation of the response to immunotherapy in the high and low agingScore groups in the E-MTAB-1980 cohort. *P < 0.05; ****P < 0.0001.

As illustrated in **Figure 9A**, the low agingScore group presented relatively high infiltrations of immunosuppressive cells (MDSCs, type 1 helper cells, type 2 helper cells, T follicular helper cells, etc.) as well as immune active cells (CD4^+^ T cells, CD8^+^ T cells, etc.) in comparison to the high agingScore group, indicating that tumors with high agingScore lacked recruitment or activation of innate immune cell populations. Moreover, the high agingScore group showed a higher expression of most co-stimulatory and co-inhibitory immune checkpoint molecules than the low agingScore group (**Figure 9B**), indicating that high agingScore might upregulate the immune checkpoint molecules to escape the immune elimination after immune activation. In **Figure 9C**, agingScore was positively linked with cancer antigen presentation, CD8^+^ T cell recruiting, NK cell recruiting, infiltration of immune cells into tumors, and killing of cancer cells. Additionally, agingScore was positively linked with stromal activation (EMT, FGFR3-related genes, angiogenesis, and WNT target) and antigen-processing machinery and was negatively linked with cell cycle progression. With the TIDE algorithm, patients who responded to immunotherapy had relatively higher agingScore both in the TCGA-KIRC cohort (**Figure 9D**) and the E-MTAB-1980 cohort (**Figure 9E**). Therefore, individuals with high agingScore had a higher probability to respond to immunotherapy.

### Post-transcriptional features correlated to agingScore

**Figure 10:**
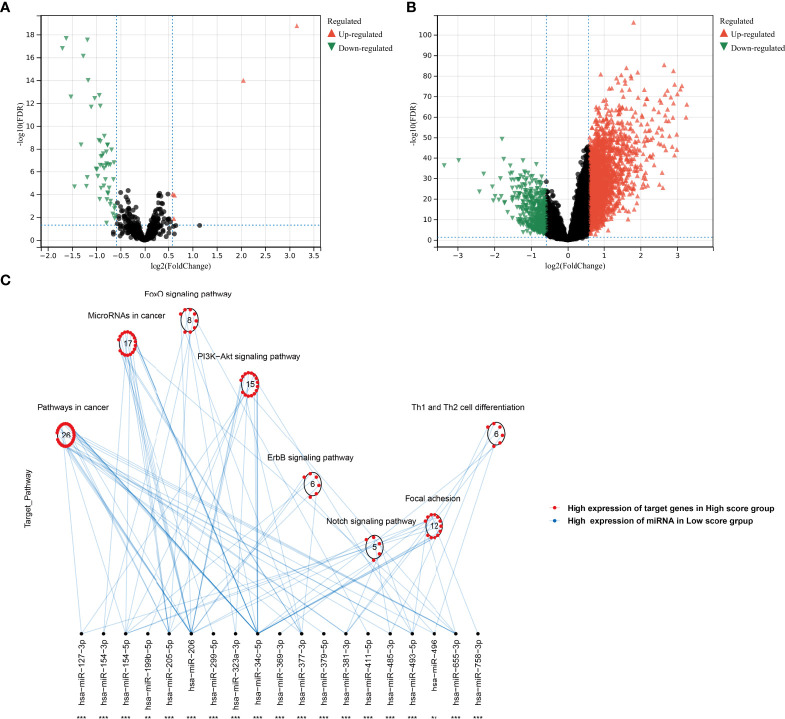
Analysis of post-transcriptional features correlated to agingScore in the TCGA-KIRC cohort. **(A)** Volcano diagram of differentially expressed miRNAs between the high and low agingScore groups. **(B)** Volcano diagram of differentially expressed RNAs between the high and low agingScore groups. Red represents upregulation, while green represents downregulation. **(C)** Differences in miRNA-targeted signaling pathways between the high and low agingScore groups. The red dot indicates the high expression of targeted mRNAs in the high agingScore group, while the blue dot indicates the high expression of miRNAs in the low agingScore group.

In the TCGA-KIRC cohort, we identified 5 upregulated miRNAs and 55 downregulated miRNAs in the high agingScore group in comparison to the low agingScore group in accordance with FDR <0.05 and at least 1.5-fold changes (**Figure 10A**). Additionally, 2,921 mRNAs were upregulated and 476 mRNAs were downregulated in the high agingScore group compared with the low agingScore group according to FDR <0.05 and at least 1.5-fold changes (**Figure 10B**). Thereafter, the targeted differentially expressed mRNAs of the above miRNAs were predicted (**Supplementary Table 5**), and enrichment analysis of the signaling pathways of their target genes was conducted. The tumorigenic pathways especially were enriched by the miRNA-targeted mRNAs in the high agingScore group, containing pathways in cancer; miRNAs in cancer; FoxO, PI3K-Akt, ErbB, and Notch signaling pathways; focal adhesion; and Th1 and Th2 cell differentiation (**Figure 10C**). These results indicated that agingScore was related to post-transcriptional mechanisms and tumorigenic pathways.

## Discussion

Over the past decade, high-throughput analysis has greatly advanced our understanding of ccRCC biology, allowing us to recapitulate critical events in ccRCC initiation and progression (2). The increase in understanding of the molecular profiles as well as genetic alterations has translated into novel targets or biomarkers, which affect ccRCC decision-making, thereby shedding a novel insight into prolonging patients’ clinical outcomes (1). Considering the unique molecular and clinical features of ccRCC, tailoring specialized management is of importance (36). Our study categorized ccRCC into three clinically and therapeutically relevant subtypes in accordance with the expression profiles of prognostic aging-associated genes (12). Additionally, agingScore was generated and presented a favorable performance in prognostication as well as immunotherapeutic responses. Our findings proposed a precise prognostic prediction approach for ccRCC patients with similar biological patterns with more precise prognostication.

ccRCC is a highly heterogeneous tumor among individual patients, encompassing different malignancies with different pathologic characteristics and molecular pathways, which makes it nearly impossible to determine a therapy that fits all ccRCC cases (4). Intratumor heterogeneity, a common feature of ccRCC, is linked to patterns of metastatic spread and prognosis after surgery, complicating the assessment of prognostic indicators (37). Hence, finding tailored therapeutic regimens for subpopulations is significant for maximizing therapeutic efficiency. In accordance with the expression profiling of 173 prognostic aging-associated genes, 529 ccRCC patients in the TCGA-KIRC cohort were classified into three aging phenotypes, which was affirmed in the E-MTAB-1980 cohort. The successful development of new immunotherapy and immunotherapy-based combination therapy requires an in-depth understanding of ccRCC immunobiology. Many chromosomal alterations are linked to the response or resistance to ICB in advanced ccRCC, and the crosstalk of somatic alterations with immune infiltrations impacts the response to ICB (38). It has been demonstrated that previous genomic correlations of ICB responses in solid tumors (TMB and PD-L1 status, etc.) cannot predict ccRCC, suggesting the important role of the immune microenvironment in modulating clinical benefits (39–41). Most ccRCC presents a moderate TMB, but high infiltration of intratumoral CD8^+^ T cells is linked to an unfavorable prognosis. Three aging phenotypes were characterized by diverse prognosis, clinical features, immune cell infiltration, tumor immunogenicity, immunotherapeutic response, and sensitivity to targeted drugs (axitinib, pazopanib, sorafenib, and sunitinib), indicating the roles of the aging process in ccRCC progression. Additionally, the interactions of the aging phenotypes with the TME reflected that aging processes seem to mediate immune cell subpopulations in ccRCC tumors.

We generated the agingScore system, which enabled us to reliably and independently predict ccRCC prognosis. Patients with low agingScore presented undesirable prognosis in contrast to those with high agingScore. Recently, Chen et al. defined cellular senescence score for delineating the cellular senescence landscape across pan-cancer, which correlated to genomic and immune features, immunotherapeutic responses, and clinical outcomes (42). The nomogram is a useful and easy-to-use tool used by doctors to predict outcomes, plan personalized therapy, and determine follow-up or imaging intervals (43). Previous research has conducted several nomogram models for ccRCC (44, 45). Regrettably, few nomograms have been applied in clinical practice. Herein, we developed the nomogram to estimate the personalized OS probability of ccRCC subjects, comprising age, stage, grade, and agingScore. Calibration plots confirmed the favorable overlap between this model-estimated and investigated OS probabilities of ccRCC. Hence, the agingScore-based nomogram may provide individualized prognosis estimates for ccRCC subjects. The agingScore was remarkably linked to tumorigenic pathways, immune cell infiltration, and tumor immunogenicity. Additionally, high agingScore patients were responsive to immunotherapy. Several small molecular compounds were determined for the low agingScore patients. Proteins showing an enhanced negative association with agingScore could possess therapeutic potential for patients with low agingScore (46). Nevertheless, most human proteins are undruggable because they are short of distinct active sites where small molecule agents can bind to as well as reside in cells that are inaccessible to biological agents. Hence, it is of importance to speculate on the potential druggable therapeutic targets for low agingScore patients with undesirable survival outcomes. Through Spearman’s correlation analysis of agingScore with the CERES score of drug targets and the expression level of druggable proteins, we determined the three genes, namely, *CYP11A1*, *SAA1*, and *GRIK4*, as promising therapeutic targets for low agingScore patients.

Collectively, our findings offered a novel insight into personalized prognostication methods as well as threw light on combining tailored risk stratification with precision treatment. Nevertheless, our analysis still possessed several limitations. At first, the number of datasets with available RNA-seq or microarray profiles remained limited. The three aging subtypes and agingScore needed to be verified with a larger sample size. Secondly, the relationships between drug targets and small molecular compounds lacked verification, thereby reducing the persuasiveness of our conclusion. Thirdly, our results were obtained from *in-silico* analysis, and more experimental and clinical verification was required to facilitate the clinical application of our findings.

## Conclusion

Our findings revealed the interactions between aging-associated genes in ccRCC and remodeling of the TME, providing a novel insight into the molecular drivers underlying ccRCC initiation and development. Collectively, our study offered opportunities for ccRCC prevention, early detection, and prognostication as well as therapy.

## Data availability statement

The datasets presented in this study can be found in online repositories. The names of the repository/repositories and accession number(s) can be found in the article/**Supplementary Material**.

## Author contributions

DM and WZ designed the study. MZ, CH, XW, YG, and XH collected the clinical information and gene expression data. MZ and CH analyzed the data and wrote the manuscript. All authors have read, revised, and approved the final manuscript.

## Funding

This study was supported by the Xiamen Medical and Health Guidance Project (3502Z202147ZD1264, 3502Z20214ZD1258, and 3502Z20214ZD1263), Fujian Provincial Science and Technology Plan Project (2021D008, 2021D010, 2021D012, and 2022D030), and Fujian Health Scientific Research Personnel Training Program (No. 2019-ZQNB-34).

## Conflict of interest

The authors declare that the research was conducted in the absence of any commercial or financial relationships that could be construed as a potential conflict of interest.

## Publisher’s note

All claims expressed in this article are solely those of the authors and do not necessarily represent those of their affiliated organizations, or those of the publisher, the editors and the reviewers. Any product that may be evaluated in this article, or claim that may be made by its manufacturer, is not guaranteed or endorsed by the publisher.

## Supplementary material

Supplementary Figure 1 Validation of three aging phenotypes across ccRCC in the E-MTAB-1980 dataset. **(A)** The CDFs of consensus matrix for k=2~9 identified by colors. **(B)** Relative alteration in area under CDFs for k=2~9. **(C)** Tracking plot for classification of TCGA-KIRC dataset into diverse subtypes for k=2~9. **(D)** Classification of the E-MTAB-1980 cohort into three clusters when k=3. **(E)** PCA of the RNA expression profiling of prognostic aging-associated genes. **(F)** Kaplan-Meier analysis for OS among three phenotypes.

Supplementary Table 1 Clinical information of TCGA-KIRC, TCGA-KIRP, and E-MTAB-1980 cohorts.

Supplementary Table 2 Univariate Cox regression analysis of 173 prognostic aging-associated genes in TCGA-KIRC dataset.

Supplementary Table 3 The list of aging phenotype-associated genes in TCGA-KIRC cohort.

Supplementary Table 4 The information of prognostic aging phenotype-associated genes in TCGA-KIRC cohort.

Supplementary Table 5 The list of targeted differentially expressed mRNAs of differentially expressed miRNAs.

## Abbreviations

RCC, renal cell carcinoma; ccRCC, clear cell renal cell carcinoma; ICB, immune checkpoint blockade; TME, tumor microenvironment; RNA-seq, RNA sequencing; TCGA, The Cancer Genome Atlas; KIRC, kidney renal clear cell carcinoma; KIRP, kidney renal papillary cell carcinoma; RMA, robust multiarray average; CAN, copy number alteration; HAGR, Human Ageing Genomic Resources; CDF, cumulative distribution function; PCA, principal component analysis; ssGSEA, single-sample gene-set enrichment analysis; GSVA, gene set variation analysis; MSigDB, Molecular Signatures Database; GO, Gene Ontology; KEGG, Kyoto Encyclopedia of Genes and Genomes; EMT, epithelial–mesenchymal transition; pan-FTBRS, pan-fibroblast TGFβ response signature; GSVA, gene set variation analysis; TIDE, Tumor Immune Dysfunction and Exclusion; CTLs, cytotoxic T lymphocytes; SubMap, Subclass Mapping; GDSC, Genomics of Drug Sensitivity in Cancer; IC_50_, half maximal inhibitory concentration; CCLs, cancer cell lines; CCLE, Cancer Cell Line Encyclopedia; CTRP, Cancer Therapeutics Response Portal; SNPs, single-nucleotide polymorphisms; SNV, single nucleotide variation; TMB, tumor mutation burden; CTAs, cancer/testis antigens; DEGs, differentially expressed genes; OS, overall survival; ROC, receiver operating characteristic; DFS, disease-free survival; DSS, disease-specific survival; PFS, progression-free survival; AUC, area under the curve; HR, hazard ratio; CI, confidence interval.

## References

[1] Bray F Ferlay J Soerjomataram I Siegel RL Torre LA Jemal A. Global cancer statistics 2018: GLOBOCAN estimates of incidence and mortality worldwide for 36 cancers in 185 countries. CA Cancer J Clin (2018) 68(6):394–424. doi: 10.3322/caac.21492 30207593

[2] Wei G Sun H Dong K Hu L Wang Q Zhuang Q. The thermogenic activity of adjacent adipocytes fuels the progression of ccRCC and compromises anti-tumor therapeutic efficacy. Cell Metab (2021) 33(10):2021–39.e8. doi: 10.1016/j.cmet.2021.08.012 34508696

[3] Jonasch E Walker CL Rathmell WK. Clear cell renal cell carcinoma ontogeny and mechanisms of lethality. Nat Rev Nephrol (2021) 17(4):245–61. doi: 10.1038/s41581-020-00359-2 PMC8172121 33144689

[4] Krishna C DiNatale RG Kuo F Srivastava RM Vuong L Chowell D. Single-cell sequencing links multiregional immune landscapes and tissue-resident T cells in ccRCC to tumor topology and therapy efficacy. Cancer Cell (2021) 39(5):662–77.e6. doi: 10.1016/j.ccell.2021.03.007 33861994 PMC8268947

[5] Banchereau R Leng N Zill O Sokol E Liu G Pavlick D. Molecular determinants of response to PD-L1 blockade across tumor types. Nat Commun (2021) 12(1):3969. doi: 10.1038/s41467-021-24112-w 34172722 PMC8233428

[6] Motzer RJ Escudier B McDermott DF George S Hammers HJ Srinivas S. Nivolumab versus everolimus in advanced renal-cell carcinoma. N Engl J Med (2015) 373(19):1803–13. doi: 10.1056/NEJMoa1510665 PMC5719487 26406148

[7] Motzer RJ Robbins PB Powles T Albiges L Haanen JB Larkin J. Avelumab plus axitinib versus sunitinib in advanced renal cell carcinoma: biomarker analysis of the phase 3 JAVELIN renal 101 trial. Nat Med (2020) 26(11):1733–41. doi: 10.1038/s41591-020-1044-8 PMC8493486 32895571

[8] Aunan JR Cho WC Søreide K. The biology of aging and cancer: A brief overview of shared and divergent molecular hallmarks. Aging Dis (2017) 8(5):628–42. doi: 10.14336/ad.2017.0103 PMC5614326 28966806

[9] Oh E Kim JH Um J Jung DW Williams DR Lee H. Genome-wide transcriptomic analysis of non-tumorigenic tissues reveals aging-related prognostic markers and drug targets in renal cell carcinoma. Cancers (Basel) (2021) 13(12):3045. doi: 10.3390/cancers13123045 34207247 PMC8234889

[10] Sato Y Yoshizato T Shiraishi Y Maekawa S Okuno Y Kamura T. Integrated molecular analysis of clear-cell renal cell carcinoma. Nat Genet (2013) 45(8):860–7. doi: 10.1038/ng.2699 23797736

[11] Gautier L Cope L Bolstad BM Irizarry RA. Affy–analysis of affymetrix GeneChip data at the probe level. Bioinformatics (2004) 20(3):307–15. doi: 10.1093/bioinformatics/btg405 14960456

[12] de Magalhães JP Costa J Toussaint O. HAGR: the human ageing genomic resources. Nucleic Acids Res (2005) 33(Database issue):D537–43. doi: 10.1093/nar/gki017 PMC539971 15608256

[13] Wilkerson MD Hayes DN. ConsensusClusterPlus: a class discovery tool with confidence assessments and item tracking. Bioinformatics (2010) 26(12):1572–3. doi: 10.1093/bioinformatics/btq170 PMC2881355 20427518

[14] Hänzelmann S Castelo R Guinney J. GSVA: gene set variation analysis for microarray and RNA-seq data. BMC Bioinf (2013) 14:7. doi: 10.1186/1471-2105-14-7 PMC3618321 23323831

[15] Yu G Wang LG Han Y He QY. clusterProfiler: an r package for comparing biological themes among gene clusters. Omics (2012) 16(5):284–7. doi: 10.1089/omi.2011.0118 PMC3339379 22455463

[16] Şenbabaoğlu Y Gejman RS Winer AG Liu M Van Allen EM de Velasco G. Tumor immune microenvironment characterization in clear cell renal cell carcinoma identifies prognostic and immunotherapeutically relevant messenger RNA signatures. Genome Biol (2016) 17(1):231. doi: 10.1186/s13059-016-1092-z 27855702 PMC5114739

[17] Rosenberg JE Hoffman-Censits J Powles T van der Heijden MS Balar AV Necchi A. Atezolizumab in patients with locally advanced and metastatic urothelial carcinoma who have progressed following treatment with platinum-based chemotherapy: a single-arm, multicentre, phase 2 trial. Lancet (2016) 387(10031):1909–20. doi: 10.1016/s0140-6736(16)00561-4 PMC5480242 26952546

[18] Mariathasan S Turley SJ Nickles D Castiglioni A Yuen K Wang Y. TGFβ attenuates tumour response to PD-L1 blockade by contributing to exclusion of T cells. Nature (2018) 554(7693):544–8. doi: 10.1038/nature25501 PMC6028240 29443960

[19] Yoshihara K Shahmoradgoli M Martínez E Vegesna R Kim H Torres-Garcia W. Inferring tumour purity and stromal and immune cell admixture from expression data. Nat Commun (2013) 4:2612. doi: 10.1038/ncomms3612 24113773 PMC3826632

[20] Chen DS Mellman I. Oncology meets immunology: the cancer-immunity cycle. Immunity (2013) 39(1):1–10. doi: 10.1016/j.immuni.2013.07.012 23890059

[21] Hoshida Y Brunet JP Tamayo P Golub TR Mesirov JP. Subclass mapping: identifying common subtypes in independent disease data sets. PloS One (2007) 2(11):e1195. doi: 10.1371/journal.pone.0001195 18030330 PMC2065909

[22] Geeleher P Cox N Huang RS. pRRophetic: an r package for prediction of clinical chemotherapeutic response from tumor gene expression levels. PloS One (2014) 9(9):e107468. doi: 10.1371/journal.pone.0107468 25229481 PMC4167990

[23] Yang W Soares J Greninger P Edelman EJ Lightfoot H Forbes S. Genomics of drug sensitivity in cancer (GDSC): a resource for therapeutic biomarker discovery in cancer cells. Nucleic Acids Res (2013) 41(Database issue):D955–61. doi: 10.1093/nar/gks1111 PMC3531057 23180760

[24] Ghandi M Huang FW Jané-Valbuena J Kryukov GV Lo CC McDonald ER 3rd. Next-generation characterization of the cancer cell line encyclopedia. Nature (2019) 569(7757):503–8. doi: 10.1038/s41586-019-1186-3 PMC6697103 31068700

[25] Mayakonda A Lin DC Assenov Y Plass C Koeffler HP. Maftools: efficient and comprehensive analysis of somatic variants in cancer. Genome Res (2018) 28(11):1747–56. doi: 10.1101/gr.239244.118 PMC6211645 30341162

[26] Mermel CH Schumacher SE Hill B Meyerson ML Beroukhim R Getz G. GISTIC2.0 facilitates sensitive and confident localization of the targets of focal somatic copy-number alteration in human cancers. Genome Biol (2011) 12(4):R41. doi: 10.1186/gb-2011-12-4-r41 21527027 PMC3218867

[27] Auslander N Zhang G Lee JS Frederick DT Miao B Moll T. Robust prediction of response to immune checkpoint blockade therapy in metastatic melanoma. Nat Med (2018) 24(10):1545–9. doi: 10.1038/s41591-018-0157-9 PMC6693632 30127394

[28] Nielsen M Andreatta M. NetMHCpan-3.0; improved prediction of binding to MHC class I molecules integrating information from multiple receptor and peptide length datasets. Genome Med (2016) 8(1):33. doi: 10.1186/s13073-016-0288-x 27029192 PMC4812631

[29] Szolek A Schubert B Mohr C Sturm M Feldhahn M Kohlbacher O. OptiType: precision HLA typing from next-generation sequencing data. Bioinformatics (2014) 30(23):3310–6. doi: 10.1093/bioinformatics/btu548 PMC4441069 25143287

[30] Chalmers ZR Connelly CF Fabrizio D Gay L Ali SM Ennis R. Analysis of 100,000 human cancer genomes reveals the landscape of tumor mutational burden. Genome Med (2017) 9(1):34. doi: 10.1186/s13073-017-0424-2 28420421 PMC5395719

[31] Thorsson V Gibbs DL Brown SD Wolf D Bortone DS Yang T-HO. The immune landscape of cancer. Immunity (2018) 48(4):812–30.e14. doi: 10.1016/j.immuni.2018.03.023 29628290 PMC5982584

[32] Ritchie ME Phipson B Wu D Hu Y Law CW Shi W. Limma powers differential expression analyses for RNA-sequencing and microarray studies. Nucleic Acids Res (2015) 43(7):e47. doi: 10.1093/nar/gkv007 25605792 PMC4402510

[33] Gao Y Wang H Li H Ye X Xia Y Yuan S. Integrated analyses of m(1)A regulator-mediated modification patterns in tumor microenvironment-infiltrating immune cells in colon cancer. Oncoimmunology (2021) 10(1):1936758. doi: 10.1080/2162402x.2021.1936758 34221700 PMC8224220

[34] Zhang B Wu Q Li B Wang D Wang L Zhou YL. m(6)A regulator-mediated methylation modification patterns and tumor microenvironment infiltration characterization in gastric cancer. Mol Cancer (2020) 19(1):53. doi: 10.1186/s12943-020-01170-0 32164750 PMC7066851

[35] Kozomara A Birgaoanu M Griffiths-Jones S. miRBase: from microRNA sequences to function. Nucleic Acids Res (2019) 47(D1):D155–d62. doi: 10.1093/nar/gky1141 PMC6323917 30423142

[36] Miao D Margolis CA Gao W Voss MH Li W Martini DJ. Genomic correlates of response to immune checkpoint therapies in clear cell renal cell carcinoma. Science (2018) 359(6377):801–6. doi: 10.1126/science.aan5951 PMC6035749 29301960

[37] Au L Hatipoglu E Robert de Massy M Litchfield K Beattie G Rowan A. Determinants of anti-PD-1 response and resistance in clear cell renal cell carcinoma. Cancer Cell (2021) 39(11):1497–518.e11. doi: 10.1016/j.ccell.2021.10.001 34715028 PMC8599450

[38] Braun DA Hou Y Bakouny Z Ficial M Sant' Angelo M Forman J. Interplay of somatic alterations and immune infiltration modulates response to PD-1 blockade in advanced clear cell renal cell carcinoma. Nat Med (2020) 26(6):909–18. doi: 10.1038/s41591-020-0839-y PMC7499153 32472114

[39] Snyder A Makarov V Merghoub T Yuan J Zaretsky JM Desrichard A. Genetic basis for clinical response to CTLA-4 blockade in melanoma. N Engl J Med (2014) 371(23):2189–99. doi: 10.1056/NEJMoa1406498 PMC4315319 25409260

[40] Riaz N Havel JJ Makarov V Desrichard A Urba WJ Sims JS. Tumor and microenvironment evolution during immunotherapy with nivolumab. Cell (2017) 171(4):934–49.e16. doi: 10.1016/j.cell.2017.09.028 29033130 PMC5685550

[41] Havel JJ Chowell D Chan TA. The evolving landscape of biomarkers for checkpoint inhibitor immunotherapy. Nat Rev Cancer (2019) 19(3):133–50. doi: 10.1038/s41568-019-0116-x PMC6705396 30755690

[42] Wang X Ma L Pei X Wang H Tang X Pei JF. Comprehensive assessment of cellular senescence in the tumor microenvironment. Brief Bioinform (2022) 23(3):bbac118. doi: 10.1093/bib/bbac118 35419596 PMC9116224

[43] Gittleman H Lim D Kattan MW Chakravarti A Gilbert MR Lassman AB. An independently validated nomogram for individualized estimation of survival among patients with newly diagnosed glioblastoma: NRG oncology RTOG 0525 and 0825. Neuro Oncol (2017) 19(5):669–77. doi: 10.1093/neuonc/now208 PMC5464437 28453749

[44] Qi-Dong X Yang X Lu JL Liu CQ Sun JX Li C. Development and validation of a nine-Redox-Related long noncoding RNA signature in renal clear cell carcinoma. Oxid Med Cell Longev (2020) 2020:6634247. doi: 10.1155/2020/6634247 33425212 PMC7781722

[45] Yin X Wang Z Wang J Xu Y Kong W Zhang J. Development of a novel gene signature to predict prognosis and response to PD-1 blockade in clear cell renal cell carcinoma. Oncoimmunology (2021) 10(1):1933332. doi: 10.1080/2162402x.2021.1933332 34262797 PMC8253123

[46] Yang C Huang X Li Y Chen J Lv Y Dai S. Prognosis and personalized treatment prediction in TP53-mutant hepatocellular carcinoma: an in silico strategy towards precision oncology. Brief Bioinform (2021) 22(3):bbaa164. doi: 10.1093/bib/bbaa164 32789496

