# Supplementary figures and images for: Aging-based molecular classification and score system in ccRCC uncovers distinct prognosis, tumor immunogenicity, and treatment sensitivity
Source: Front Immunol. 2022 Aug 11;13:877076. doi: 10.3389/fimmu.2022.877076 (PMC9402984; doi:10.3389/fimmu.2022.877076)

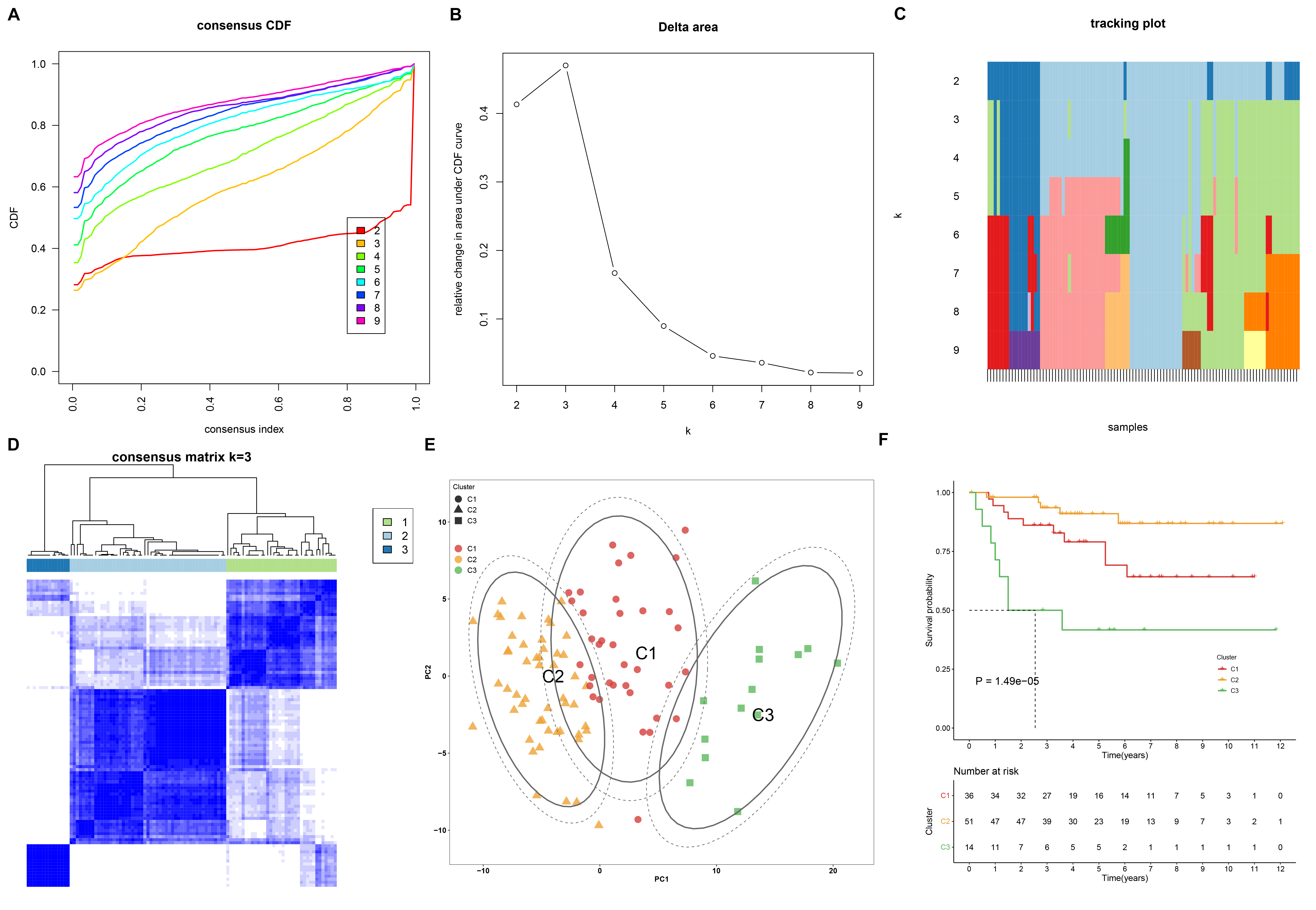

Supplement: Supplementary Figure 1 — Validation of three aging phenotypes across ccRCC in the E-MTAB-1980 dataset. (A) The CDFs of consensus matrix for k=2~9 identified by colors. (B) Relative alteration in area under CDFs for k=2~9. (C) Tracking plot for classification of TCGA-KIRC dataset into diverse subtypes for k=2~9. (D) Classification of the E-MTAB-1980 cohort into three clusters when k=3. (E) PCA of the RNA expression profiling of prognostic aging-associated genes. (F) Kaplan-Meier analysis for OS among three phenotypes. [file Image_1.tif]
